# Abundance and Localization of Symbiotic Bacterial Communities in the Fly Parasitoid Spalangia cameroni

**DOI:** 10.1128/aem.02549-21

**Published:** 2022-04-14

**Authors:** Sarit Rohkin Shalom, Benjamin Weiss, Maya Lalzar, Martin Kaltenpoth, Elad Chiel

**Affiliations:** a Department of Biology and Environment, Faculty of Natural Sciences, University of Haifa -Oranim, Tivon, Israel; b Department for Evolutionary Ecology, Institute of Organismic and Molecular Evolution, Johannes Gutenberg University, Mainz, Germany; c Bioinformatics Service Unit, University of Haifa, Haifa, Israel; d Department of Insect Symbiosis, Max Planck Institute for Chemical Ecology, Jena, Germany; University of Queensland

**Keywords:** *Arsenophonus*, *Sodalis*, *Rickettsia*, *Wolbachia*, community ecology, qPCR

## Abstract

Multicellular eukaryotes often host multiple microbial symbionts that may cooperate or compete for host resources, such as space and nutrients. Here, we studied the abundances and localization of four bacterial symbionts, *Rickettsia, Wolbachia*, *Sodalis*, and *Arsenophonus*, in the parasitic wasp Spalangia cameroni. Using quantitative PCR (qPCR), we measured the symbionts’ titers in wasps that harbor different combinations of these symbionts. We found that the titer of each symbiont decreased as the number of symbiont species in the community increased. Symbionts' titers were higher in females than in males. *Rickettsia* was the most abundant symbiont in all the communities, followed by *Sodalis* and *Wolbachia*. The titers of these three symbionts were positively correlated in some of the colonies. Fluorescence *in situ* hybridization was in line with the qPCR results: *Rickettsia, Wolbachia*, and *Sodalis* were observed in high densities in multiple organs, including brain, muscles, gut, Malpighian tubules, fat body, ovaries, and testes, while *Arsenophonus* was localized to fewer organs and in lower densities. *Sodalis* and *Arsenophonus* were observed in ovarian follicle cells but not within oocytes or laid eggs. This study highlights the connection between symbionts’ abundance and localization. We discuss the possible connections between our findings to symbiont transmission success.

**IMPORTANCE** Many insects carry intracellular bacterial symbionts (bacteria that reside within the cells of the insect). When multiple symbiont species cohabit in a host, they may compete or cooperate for space, nutrients, and transmission, and the nature of such interactions would be reflected in the abundance of each symbiont species. Given the widespread occurrence of coinfections with maternally transmitted symbionts in insects, it is important to learn more about how they interact, where they are localized, and how these two aspects affect their co-occurrence within individual insects. Here, we studied the abundance and the localization of four symbionts, *Rickettsia, Wolbachia*, *Sodalis*, and *Arsenophonus*, that cohabit the parasitic wasp Spalangia cameroni. We found that symbionts’ titers differed between symbiotic communities. These results were corroborated by microscopy, which shows differential localization patterns. We discuss the findings in the contexts of community ecology, possible symbiont-symbiont interactions, and host control mechanisms that may shape the symbiotic community structure.

## INTRODUCTION

Most, if not all, insects interact with a variety of microbes, and the nature of the interactions span from obligate mutualism to parasitism (1). Some of these microbes are bacteria that reside within specialized cells (bacteriocytes) of their host and synthesize nutrients and are therefore critical for the host’s development and reproduction. Such bacteria are commonly termed “primary” or “obligate” symbionts. Other symbiotic intracellular bacteria, commonly termed “facultative” or “secondary” symbionts, do not have a nutritional role but can enhance the host’s survival by providing defense against natural enemies or protection to abiotic stressors. A few lineages of facultative symbionts (FS) manipulate the reproduction of their host to produce more females, which transmit the symbiont(s) to their offspring (2, 3). Both obligate and facultative symbionts are maternally transmitted, but FS can also be horizontally transferred (3, 4).

When multiple symbionts co-occur within the same host, the host can be viewed as a habitat in which the symbionts likely compete for the (limited) host resources—space and nutrients—by a variety of mechanisms, such as secreting antimicrobial compounds (interference competition), depleting host resources (exploitative competition), or inducing a host immune response that is deleterious to the competitors (5–8). The host may suffer higher fitness costs if the symbionts overexploit its resources; therefore, hosts also employ mechanisms for regulating the titers of their symbionts (5, 9, 10, 11). On the other hand, such fitness costs may be compensated by the coexisting symbionts if they confer fitness advantage(s) to their host. The abundance of symbionts is linked to their effects and transmission: low symbiont titers may result in incomplete vertical transmission, whereas high titers may cause negative effects on their host (9). The interactions between the symbionts may also depend on their cohabitation in organs, cells, or bacteriocytes. Thus, the symbiotic community will vary according to the ecological selection pressures that operate in each environment, leading to either fixation of different symbiotic communities in different environments or the occurrence of various symbiotic communities within a population (12, 13).

Here, we studied the (quantitative) community structure and the localization of FS of the parasitic wasp Spalangia cameroni Perkins (Hymenoptera: Pteromalidae). This wasp is a natural enemy of filth flies (Diptera: Muscidae), including major pest species such as the house fly, Musca domestica, and the stable fly, Stomoxys calcitrans, and is one of a few species that have been commercialized as biocontrol agents. The wasp lays its eggs onto fly pupae (most often a single egg per pupa), which serve as the sole food source for the developing wasp larvae. Four lineages of FS have been found in S. cameroni, *Rickettsia* (R), *Wolbachia* (W) (both of the order *Rickettsiales*), *Sodalis* (S), and *Arsenophonus* (A) (both of the order *Enterobacterales*) (14–16). *Wolbachia* is a ubiquitous symbiont of arthropods and nematodes, famous for manipulating the reproduction of its hosts, but in some hosts, it has a nutritional or a protective role (3, 17). Spalangia cameroni harbors multiple *Wolbachia* strains that collectively cause an incomplete cytoplasmic incompatibility (14, 16, 18, 19). The *Rickettsia* of S. cameroni clusters to the “transitional group” (16), which consists of members from diverse hosts, such as the pathogenic Rickettsia felis from fleas, as well as nonpathogenic *Rickettsia* from assorted other hosts (20). Nonpathogenic *Rickettsia* was found to be reproductive manipulators, causing parthenogenesis in some hymenopterans (21–23) and male killing in some coccinellid beetles (24, 25). In *Spalangia endius*, a congener of S. cameroni, *Rickettsia* does not cause reproductive manipulations but has mild deleterious effects on its fitness (26). The *Sodalis* of S. cameroni is most closely related to S. praecaptivus (a free-living bacterium species) and to amino acid-provisioning symbionts of several weevils (16). *Sodalis* spp. have not been reported to cause reproductive manipulations. *Arsenophonus* is also a common clade of insect symbionts, including some species of filth fly parasitoids, known to cause male killing in the parasitoid Nasonia vitripennis (15, 27). The effects of *Sodalis* and *Arsenophonus* on S. cameroni are yet to be studied.

Given the widespread occurrence of coinfections with maternally transmitted symbionts in insects, it is important to learn more about how they interact, where they are localized, and how these two aspects affect their co-occurrence within individual insects. In the current study, we established S. cameroni colonies carrying various combinations of single or multiple FS, measured the titer of each symbiont in each colony, and studied their localization. Our hypotheses were (i) tissue tropism affects the interactions between co-occurring symbionts and their abundance in co-occurrence versus single infections, and (ii) *Wolbachia*, being fixed in all the colonies, will be the predominant symbiont in the communities.

## RESULTS

### Symbionts' titers.

**FIG 1:**
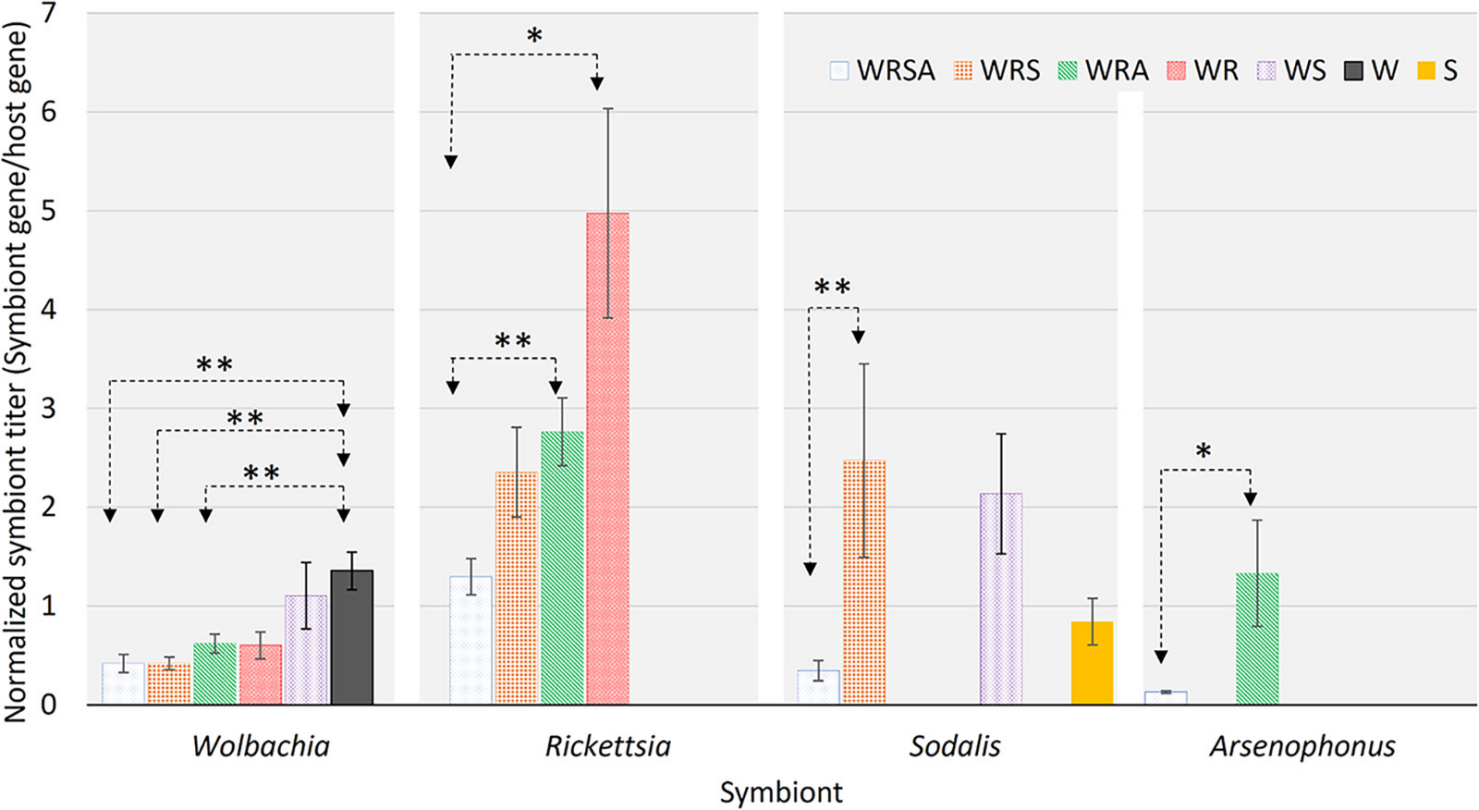
Normalized symbiont titers (means ± standard errors) in S. cameroni colonies with different combinations of intracellular symbionts (females and males combined). Significant statistical differences between colonies are denoted by asterisks (*, *P* < 0.05; **, *P* < 0.01; Wilcoxon rank-sum exact test). W, *Wolbachia*; R, *Rickettsia*; S, *Sodalis*; A, *Arsenophonus*.

**FIG 2:**
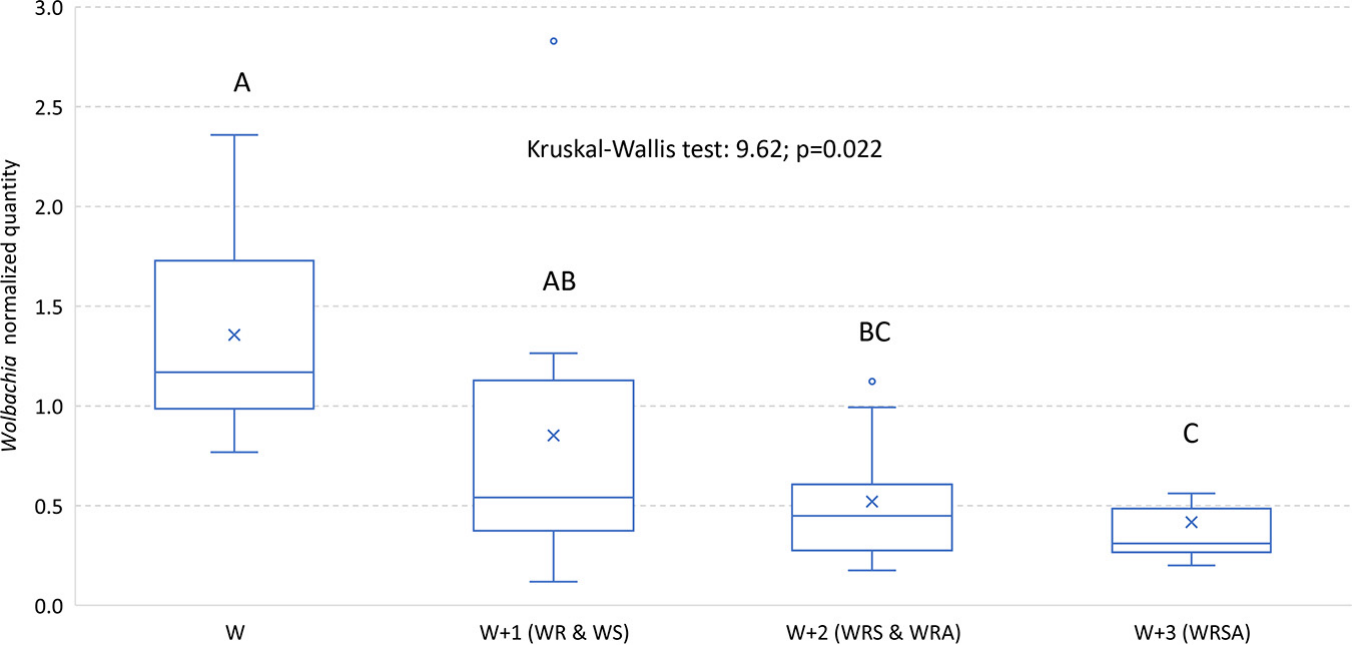
Normalized titers of *Wolbachia* in S. cameroni when it is the only symbiont (W), when there is one additional symbiont (W + 1 = WR and WS), two additional symbionts (W + 2 = WRS and WRA), and three additional symbionts (W + 3 = WRSA). The inner horizontal line in each box is the median; “X” denotes the average. The groups were compared by nonparametric tests (see the text for details).

**FIG 3:**
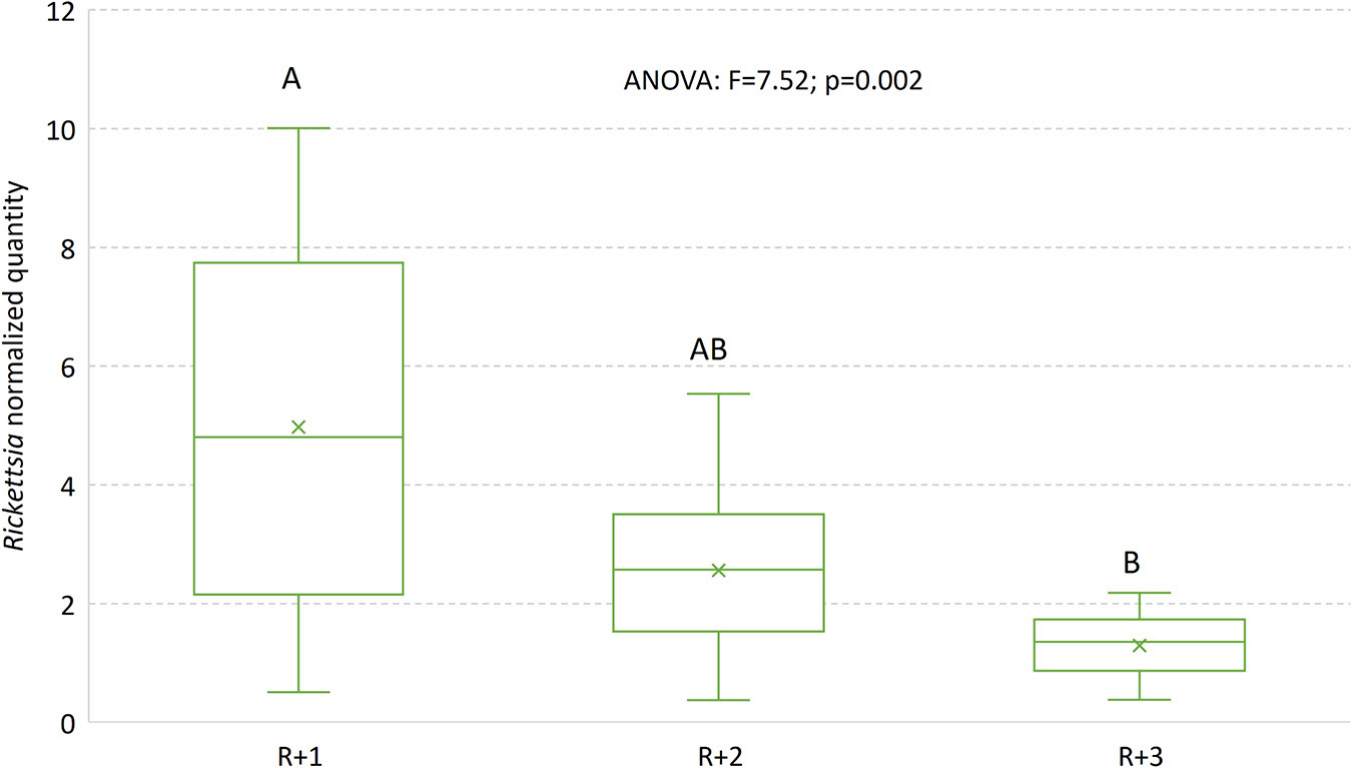
Normalized titers of *Rickettsia* in S. cameroni when there is one additional symbiont (*R* + 1 = WR), two additional symbionts (*R* + 2 = WRS and WRA), and three additional symbionts (*R* + 3 = WRSA). The inner horizontal line in each box is the median; “X” denotes the average. The one-way ANOVA was performed after log(X + 1) transformation to meet the parametric test preconditions.

*Rickettsia* was the most abundant symbiont, comprising between 45% (WRS) to 89% (WR) of the communities (Fig. 1). The titers of *Wolbachia* and *Rickettsia* decreased as the community included more members (Table 1; Fig. 1). The same tendency—decreases in *Wolbachia* and *Rickettsia* quantities as the community included more members—was observed when the data of double- and triple-infected colonies were pooled (Fig. 2 and 3). *Arsenophonus* titer was higher in the WRA colony than in the WRSA colony (*P = *0.07). *Sodalis* titer was lowest in the WRSA colony, highest in the WRS and WS colonies, and intermediate in the singly infected S colony. The following symbionts were positively and significantly correlated: *Wolbachia-Rickettsia* (only in the WRA colony), *Wolbachia-Sodalis* (only in the WS colony), and *Rickettsia-Sodalis* (only in the WRS colony) (Table 2).

**TABLE 1:**
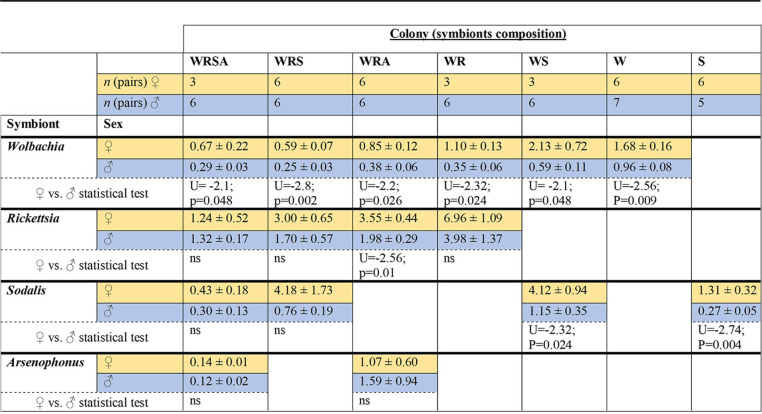
Normalized titers of *Wolbachia*, *Rickettsia*, *Sodalis*, and *Arsenophonus* in colonies of Spalangia cameroni^*a*^

a Values are averages ± standard errors. Results were subjected to Mann-Whitney tests. W, *Wolbachia*; R, *Rickettsia*; S, *Sodalis*; A, *Arsenophonus*; ns, not significant.

**TABLE 2:** Result of Pearson correlation coefficient tests between the titers of the four symbionts in each colony separately^*a*^

Colony	Symbiont 1	Symbiont 2	Pearson *r* value (df)	*P* value
WRSA	*Rickettsia*	*Arsenophonus*	0.232 (7)	0.55
WRA	*Rickettsia*	*Arsenophonus*	−0.090 (11)	0.78
WRSA	*Rickettsia*	*Sodalis*	0.408 (7)	0.27
WRS	*Rickettsia*	*Sodalis*	0.774 (11)	**0.003**
WRSA	*Sodalis*	*Arsenophonus*	0.586 (7)	0.09
WRSA	*Wolbachia*	*Arsenophonus*	0.122 (7)	0.75
WRA	*Wolbachia*	*Arsenophonus*	−0.226 (11)	0.48
WRSA	*Wolbachia*	*Rickettsia*	0.532 (7)	0.14
WR	*Wolbachia*	*Rickettsia*	0.629 (7)	0.07
WRA	*Wolbachia*	*Rickettsia*	0.702 (11)	**0.0108**
WRS	*Wolbachia*	*Rickettsia*	0.450 (11)	0.14
WRSA	*Wolbachia*	*Sodalis*	0.392 (7)	0.29
WRS	*Wolbachia*	*Sodalis*	0.469 (11)	0.12
WS	*Wolbachia*	*Sodalis*	0.939 (11)	**0.0002**

a Statistically significant pairs are in bold.

### Differences between sexes.

Endosymbionts’ titers were generally higher in females than in males (Table 1), most notably in *Wolbachia*, with roughly 2-fold statistically significant differences in all the colonies. *Sodalis* titers were also higher in females than males, especially in the S and WS colonies. *Rickettsia* titers were significantly higher in females only in the WRA colony. For *Arsenophonus*, no significant differences were found between the sexes. A table of the full factorial comparisons between colonies, symbionts, and sexes is available in supplementary file 2.

### Localization of the symbionts.

**FIG 4:**
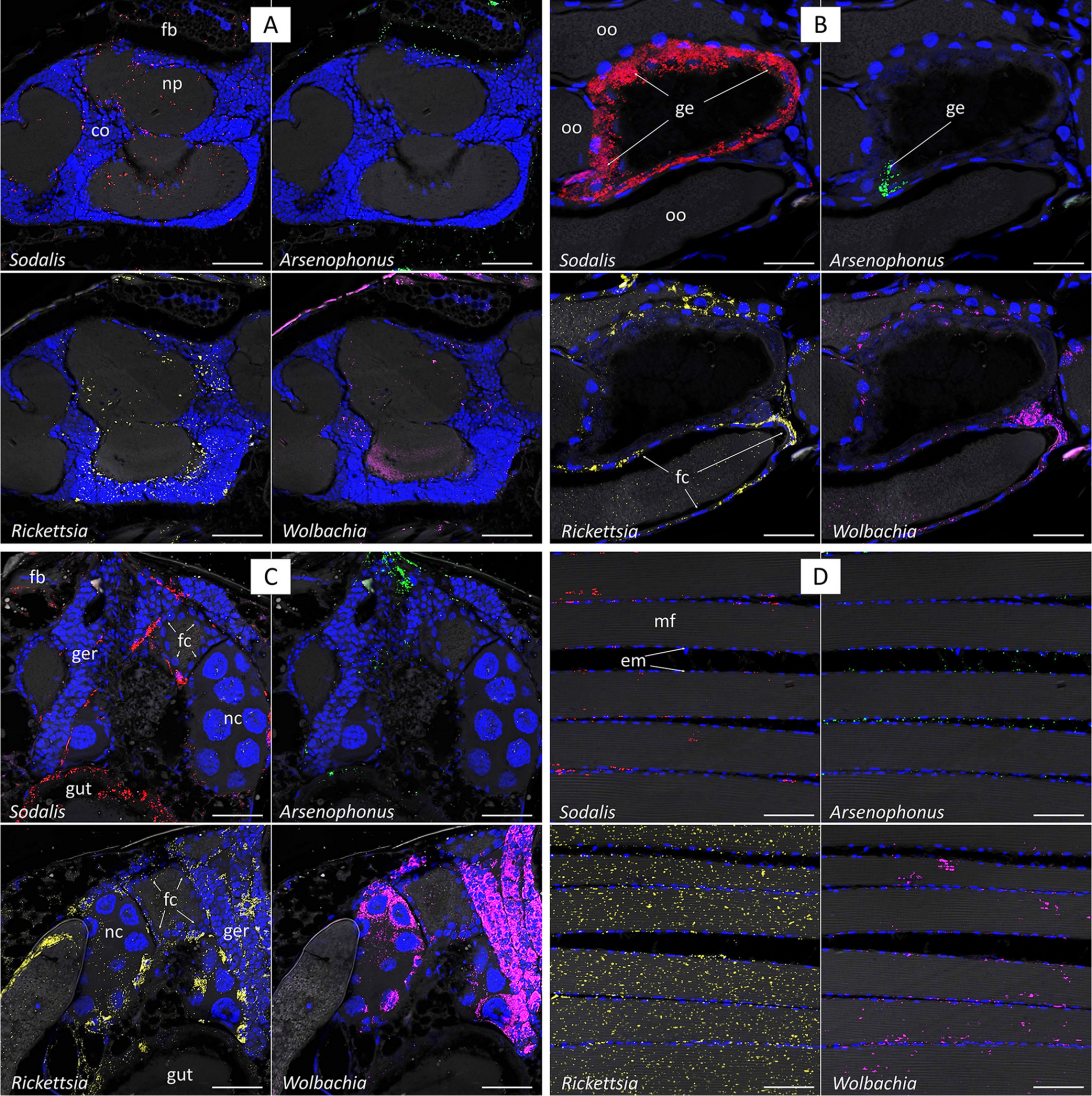

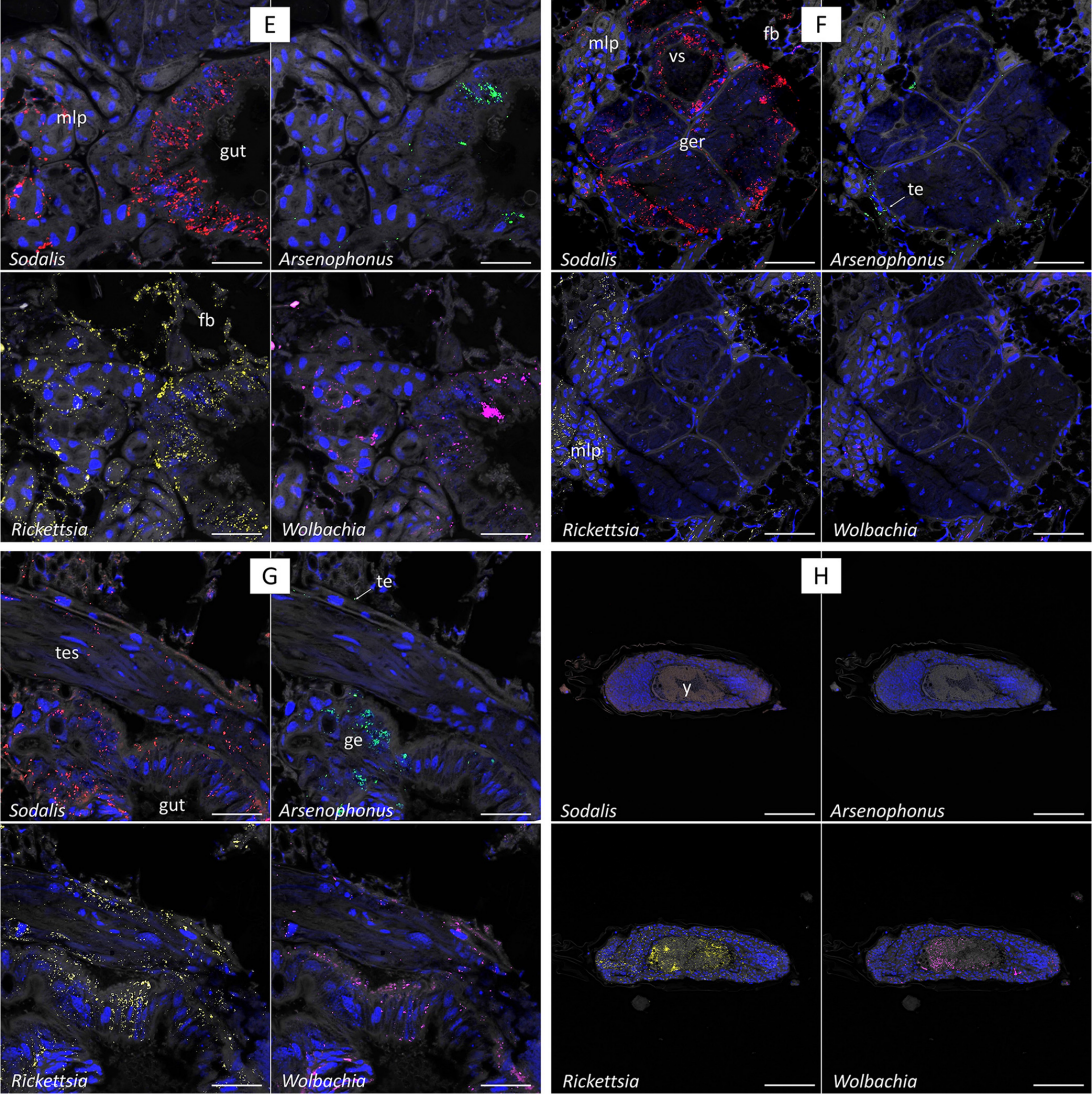
Localization of the four symbionts in various tissues of S. cameroni. (A) Brain (co, cortex; np, neuropil; fb, fat body); (B) gut and oocytes (oo, oocyte; ge, gut epithelium; fc, follicle cells); (C) ovaries (nc, nurse cells; fc, follicle cells; ger, germarium; fb, fat body); (D) flight muscles (mf, muscle fibers; em, endomysium); (E) Malpighian tubules (mlp); (F) testes (vs, vesicula seminalis; ger, germarium; mlp, Malpighian tubules; te, testicular epithelium); (G) testis maturation and transformation zone (ge, gut epithelium; te, testicular epithelium; tes, testicles); (H) 24- to 48-h-old laid eggs (y, yolk). Scale bar, 50 μm.

All the four symbionts were found in the following tissue, albeit in different densities (Table 3): gut epithelia, fat body, Malpighian tubules, thoracic flight muscles, and ovaries. Notably, in the ovaries, *Arsenophonus* and *Sodalis* were found in low densities only in follicle cells and in the germarium, but not within the oocytes, whereas *Rickettsia* and *Wolbachia* were detected in much higher densities in the oocytes, nurse cells, germarium, and follicle cells (Fig. 4C). Likewise, in laid eggs, only *Wolbachia* and *Rickettsia* were detected, while *Arsenophonus* and *Sodalis* were not (Fig. 4H). In the male reproductive tissues, *Wolbachia*, *Rickettsia*, and *Sodalis* were detected in the testes’ maturation and transformation zone (most abundantly *Rickettsia*; Fig. 4G); *Sodalis* was additionally detected in the germarium and the seminal vesicles (Fig. 4F); *Arsenophonus* was detected in minute densities only in the testicular epithelium (Fig. 4G and H); and *Rickettsia*, *Wolbachia*, and *Sodalis* were detected in the cortex and neuropil of the brain (Fig. 4A). Among the four symbionts, the symbiont that appeared in the highest densities in most of the tissues was *Rickettsia*, especially in the thoracic flight muscles (Fig. 4D). *Wolbachia* was numerous in the ovaries, and *Sodalis* was abundant in the gut epithelia. *Arsenophonus* was the least abundant symbiont in all the tissues.

**TABLE 3:** Summary of localization and approximate abundance of the four symbionts in various organs of their host, S. cameroni^*a*^

Organ	*Wolbachia*	*Rickettsia*	*Sodalis*	*Arsenophonus*
Brain				
Cortex	+	+	+	−
Neuropil	+	++	++	−
Flight muscles				
Endomysium	+	+	+	+
Fibers	++	+++	+	−
Gut				
Epithelia	++	++	+++	+
Lumen	−	−	−	−
Malpighian tubules	++	++	++	−/+
Fat body	+	++	++	++
Ovaries				
Germarium	++++	+++	−/+	−/+
Nurse cells	+++	++	−	−
Follicle cells	++	++	−/+	−/+
Oocytes	++	++	−	−
Laid eggs	++	+++	−	−
Testicles				
Germarium	−	−	+	−
Maturation and transformation zone	−	−	+	−
	−	−	−/+	−
Epithelium	−	+	++	+

a −, not detected; −/+, detected in very low density; +, low density; ++, intermediate density; +++, high density; ++++, very high density.

## DISCUSSION

In this research, we studied the abundance and localization of FS of the parasitoid S. cameroni. The results show that symbionts' titers change substantially between wasps with different symbiotic composition. Symbiotic communities are shaped by the nature of the interactions between the symbionts (bottom-up control), by host control measures (top-down control), as well as by extrinsic selection factors (environmental conditions, hosts’ natural enemies, and more), collectively termed in reference 28 as an “ecosystem on a leash model.” Theory predicts that in beneficial interactions, natural selection will favor an increase in symbionts’ abundance as long as the benefits to the host are higher than the costs of maintaining the symbionts (28, 29). High symbiont titers do not necessarily translate to higher benefits to the host. For example, the protection against viruses conferred by *Wolbachia* is correlated with its titers in Drosophila simulans (30, 31), whereas variations in titers of *Regiella* have a minor effect on the protection it confers to its pea aphid host against pathogenic fungi (32). Another notable result is that symbionts’ titers were higher in females than in males (Table 1). This makes sense because these symbionts are transmitted maternally only, and high symbiont titers in females are important for the success of vertical transmission to the offspring. For example, low densities of Regiella insecticola, an FS of the pea aphid, leads to the symbiont’s complete loss (32). Nonetheless, while high symbiont titers correlate with high fidelity of vertical transmission, excessive symbiont titers can be costly to the host and lead to decreased transmission rates (because the host may produce less offspring or die early) (32). Hence, in that respect, too, titers are expected to stabilize around an equilibrium point between the costs and benefits of both the host and the symbionts (host- and symbiont-level selection, respectively). High symbiont titers are important for successful horizontal transmission as well (33, 34).

*Wolbachia* is fixed in field populations of S. cameroni (16), suggesting that *Wolbachia* has a longstanding interaction with this host. Early-arriving species may encumber the establishment of additional symbionts (priority effects) (28), but apparently, this is not the case here. *Wolbachia* is a well-known reproductive manipulator in many arthropods; in S. cameroni, it induces cytoplasmic incompatibility (CI) (14), a manipulation that may have led to the fixation of *Wolbachia* in S. cameroni populations. The high density of *Wolbachia* in the reproductive tissues is thus not surprising, even though the correlation between CI strength and *Wolbachia* titers is variable (9, 35). *Wolbachia* in S. cameroni is localized in multiple organs and tissues (Fig. 4), which is common in many other hosts (36) but surprisingly different from the congener S. endius, in which *Wolbachia* was found to be restricted to the ovaries (26). The reason for this is unknown; perhaps different *Wolbachia* strains differ in their tissue tropism. So far, we do not know whether *Wolbachia* benefits S. cameroni in some way, such as pathogens or insecticide resistance, as has been reported for *Wolbachia* and/or *Rickettsia* in some host taxa, although there are also opposite reports of increased insecticide susceptibility (3, 37).

In the following section, we discuss the relative titers of the symbionts. We acknowledge that the amplification efficiency differs between the primer pairs used specifically for each symbiont (although the qPCR software corrects the output accordingly), and therefore, we interpret these results with caution. *Rickettsia* dominates the microbial community in S. cameroni in terms of relative titers (Fig. 1), as can also be seen in the fluorescence *in situ* hybridization (FISH) images (Fig. 4), suggesting that *Rickettsia* may be less sensitive to immune effectors of S. cameroni. Concordantly, it was recently found that R. parkeri evades autophagy and ubiquitylation due to methylation of lysins in the outer membrane protein B (38). The *Rickettsia* in S. cameroni is closely related to the pathogenic species R. felis (16), providing a possible explanation to the high titers and the pervasive tissue tropism of this symbiont, resembling the tissue tropism of R. felis in the cat flea (39). A similar pattern of a *Rickettsia*-dominated community was recently found also in whiteflies (40). Interestingly, *Rickettsia* levels in whiteflies are positively correlated with the levels of vitellogenin (41), highlighting another determinant of symbionts’ titers. The effects of *Rickettsia* on the fitness of S. cameroni are yet to be studied. In the congener S. endius, *Rickettsia* causes a mild developmental delay and does not induce reproductive manipulations (26). *Rickettsia* is highly prevalent in S. cameroni field populations, but in the location where the parasitoids were collected for the current study, *Rickettsia* always occurs together with *Wolbachia* (16). Taken together, a likely scenario is that *Rickettsia* had inhabited S. cameroni after *Wolbachia* and is spreading in S. cameroni populations either by taking advantage of the *Wolbachia*-induced CI or by benefitting S. cameroni in some way. *Wolbachia* and *Rickettsia* were found to cohabit various host species, for example, whiteflies (42), bugs (43), weevils (44), and aphids (45).

This microecosystem becomes more complex when *Sodalis* and *Arsenophonus* join the community. The titers of *Sodalis* in the WS and WRS colonies were notably higher (but not statistically significant) than in the S colony, which could be because *Wolbachia* is facilitating *Sodalis*. In the quadruple-infected colony (WRSA), where both *Arsenophonus* and *Sodalis* are present, their titers are substantially lower than in other colonies, reflecting a possible antagonism between the two. *Arsenophonus* spp. and *Sodalis* spp. co-occur in hippoboscid flies, functioning either as obligate or facultative symbionts (46, 47). Hosts can curb their symbionts’ (enthusiasm) population growth by secreting antimicrobial peptides (AMPs); similarly, symbionts can produce antibiotics and bacteriocins to eliminate competitors (28). *Sodalis* spp. employ several virulence factors, such as the PhoP/PhoQ system utilized to resist host AMPs during colonization (48) and type III secretion system (49–52), which may play a role in host infection/persistence as well as symbiont-symbiont interactions in our system as well. Further studies employing transcriptomics and proteomics approaches may shed a light on the mechanisms employed by the four community members.

The WRA, WS, and S colonies were never found in the field; they “evolved” in the lab: the first two were originally WRSA, which have lost *Sodalis* (hence, WRA) or *Rickettsia* and *Arsenophonus* (hence WS); the S colony was generated during an experiment of horizontal transmission of WRS in which only *Sodalis* successfully established (53). This highlights that the symbiotic composition is dynamic and is dictated by selection pressures that differ between field and laboratory conditions (54). Additionally, there might be certain host and symbiont genotypes that are more prone to facilitate interactions with other symbionts. The symbiotic community in the WRSA colony is quite unstable under our lab rearing conditions, frequently “losing” *Arsenophonus* and/or *Sodalis* (Sarit R.S. and Elad C., unpublished data). Our findings provide a mechanistic explanation to this phenomenon: both symbionts are present in low densities in the ovaries and were not detected within the oocytes (Fig. 4B, C, and H). Perhaps these two symbionts are transmitted like *Arsenophonus* in Nasonia vitripennis (a fly parasitoid from the same family of *Spalangia*, Pteromalidae): *Arsenophonus* is external to the oocyte, it is injected onto the fly host with the eggs, and the larvae acquire it by feeding throughout their development (55). Interestingly, all four symbionts were detected in the testicles, most prominently *Rickettsia*; *Sodalis* is the only symbiont among the four in our system that is localized also inside the seminal vesicles, suggesting the potential for paternal transmission as was reported in tsetse flies (56). Nonetheless, none of these symbionts is transmitted paternally in our model system (data not shown). Taken together, symbionts may compete for transmission, meaning that overall symbiont titers may not be the most important factor. Rather, colonizing the relevant organs (ovaries, possibly testes) and increasing the chances to infect the offspring would be essential; hence, competition for these spaces takes place. The transmission route of *Arsenophonus* in *Nasonia* is particularly interesting in this respect, as it may circumvent competition for the ovaries.

To conclude, the study system of S. cameroni and its four FS provides unique information on symbiont-host interactions and indicates a connection between symbionts’ abundance, localization, and transmission.

## MATERIALS AND METHODS

### Insect rearing.

**(i) House flies.** Adult house flies were held in net cages with water and a diet of sugar, milk powder, and egg yolk powder mixture (2:2:1 by weight, respectively). The larvae were reared on a medium of wheat bran mixed with calves' food pellets and wetted with water to 60 to 65% moisture. The flies were maintained at 26 ± 1°C, 60% ± 20% relative humidity (RH), and 14 h photophase. The flies were tested by diagnostic PCR and were found to be free of the wasps’ endosymbionts.

**(ii) Parasitoids.** Spalangia cameroni was collected in 2015 from an egg-laying poultry facility in Hazon, Israel (32°54′25.8″N, 35°23′49.0″E) using sentinel pupae as described in reference 57. The parasitoids that emerged from the sentinel pupae were separated into isofemale lines: using a fine brush, each female parasitoid was placed individually in a plastic cup (30 cm^3^ volume, with a perforated lid to allow ventilation) with 50 house fly pupae (48 h old) for oviposition for 3 days and then retrieved, identified to the species level (57, 58), and symbiont infection was determined by testing two of the emerging offspring in each cup by PCR, as described in reference 18. Subsequently, wasps with identical infection status were pooled to establish the following colonies (W, *Wolbachia*; R, *Rickettsia*; S, *Sodalis*; A, *Arsenophonus*): WRSA (2 founders), WRS (3 founders), WR (14 founders), and W (12 founders). The parasitoids were subsequently reared on house fly pupae under conditions of 26°C ± 1°C, 60% ± 20% RH, and 14 h photophase. The WRA, WS, and S colonies “evolved” in the lab: the first two were originally WRSA, which subsequently lost *Sodalis* (hence WRA) or *Rickettsia* and *Arsenophonus* (hence WS); the S colony was generated during an experiment of horizontal transmission of WRS into an uninfected line in which only *Sodalis* successfully established (53) (the number of founders of WRA, WS, and S was not recorded).

### Sample collection and lysate preparation.

Zero- to 24-h-old wasps were collected from all seven colonies and stored at −20°C. Each wasp was ground with a sterile plastic pestle in 25 μL of lysis solution (10 mM Tris-HCl, pH 8.2, 1 mM EDTA, and 25 mM NaCl) containing 2 mg mL^−1^ proteinase K (VWR, OH, USA). The lysates were incubated for 20 min at 60°C and then 10 min at 95°C and kept at −20°C until further use. Each sample was verified by diagnostic PCRs to have the expected symbionts (Table 4). In order to have sufficient sample volume for all qPCR tests, every two samples from the same colony (i.e., the same infection status) and sex were pooled into one sample. We generated at least 6 replicates (i.e., 12 wasps, 2 per sample) from each sex from each colony.

**TABLE 4:** Details of qPCR primers and conditions used in our study

Target organism	Gene	Primer sequence (5′→3′)^*f*^	Product length (bp)	Primer final concn (nM)^*c*^	Annealing temp (°C)	GenBank accession no. (reference)^*a*^^,^^*b*^
Spalangia cameroni ^*d*^	28S	F, GTGAAACCGTTCAGGGGTAA	86	400	60	AY855180.1
		R, GATTCCAAGCAAGAGCCAAC	86	400	60	AY855180.1
*Wolbachia* ^*d*^	wsp	F, AACAGCAATTTCAGGGCTAGTT	121	400	60	AF288988.1
		R, AGCGTCTTTCAAAGGAGTGC	121	400	60	AF288988.1
*Rickettsia* ^*d*^	gltA	F, TCGCAAATGTTTACGGTACTTT	74	300	60	MF041980.1 (62)
		R, TCGTGCATTTCTTTCCACTGCG	74	300	60	MF041980.1 (62)
*Sodalis* ^*d*^	ompA	F, ACCCGTCTGGACTACCAGTG	85	400	60	MF041981.1
		R, CAACGCTCAGCATGGAGTTA	85	400	60	MF041981.1
*Arsenophonus* ^*e*^	infB	F, CAACACCACTTGCCATACCA	142	300	60	HM594708.1
		R, GAAGAGTGGGGTGGTGAAAA	142	300	60	HM594708.1

a Primers were designed based on these accession numbers.

b Reference was incorporated in our primers’ design.

c For diagnostic PCR, the final concentration was 1 μM.

d qPCR conditions were 20 s at 95°C, 37 cycles of 3 s at 95°C and 20 s at 60°C, followed by 15 s at 95°C, 60 s at 60°C, and 15 s at 95°C.

e qPCR conditions were 60 s at 95°C, 37 cycles of 15 s at 95°C and 60 s at 60°C, followed by 15 s at 95°C, 60 s at 60°C, and 15 s at 95°C.

f F, forward; R, reverse.

### Quantitative PCR.

A single-copy gene fragment was chosen and amplified for each of the symbionts, *Wolbachia* surface protein (*wsp*), *Rickettsia* citrate synthase (*gltA*), *Sodalis* outer membrane protein A (*ompA*), and *Arsenophonus* translation initiation factor (*infB*). A fragment of the S. cameroni 28S rRNA gene was amplified for normalizing the data. The gene fragments were synthesized and cloned into 2,710-bp Puc57 plasmids (Bio Basic Inc., Canada) with a single gene fragment per plasmid and were then linearized using the HindIII-HF restriction enzyme (New England Biolabs, MA, USA). A standard curve was constructed for each of the five target genes using serial dilutions of the linearized plasmids (the copy numbers of the initial concentrations were calculated using the calculator in http://www.scienceprimer.com/copy-number-calculator-for-realtime-pcr). qPCR was performed using the Step One Plus real-time PCR system (Applied Biosystems, CA, USA) and Fast SYBR green master ×2 (Thermo Fisher Scientific, MA, USA). For each 20-μL reaction mixture, 1 μL of sample lysate was used, and each sample was measured in triplicates to ensure technical accuracy. Titers of the amplified gene fragments were calculated by the StepOne software v2.3 (Thermo Fisher Scientific, MA, USA). PCR conditions, primers, etc., are detailed in Table 1, and the standard curves’ parameters are detailed in Table S1 in the supplemental material.

### Statistical analysis.

The data were first normalized ($\text{symbiont titer}=\frac{\text{symbiont quantity}}{\text{wasp }28\text{S quantity}}$). We then analyzed the differences in the titers of each symbiont between the colonies using Wilcoxon rank-sum exact test. To account for the effects of repeated testing on test statistical significance levels, we applied the Holm correction. We also tested the correlations between the symbionts' titers using the Pearson correlation test and analyzed the differences in symbionts' titers between females and males (for each symbiont and colony separately) using Mann-Whitney tests.

### Determining the symbionts’ localization by fluorescence *in situ* hybridization.

Adult females, males, and eggs of S. cameroni from the WRSA colony were fixed in 4% formaldehyde (in 1× phosphate-buffered saline [PBS]) for 24 h, washed with 80% ethanol (30 min × 3 times), and then embedded in Technovit 8100 (Heraeus Kulzer, Wehrheim, Germany). Semithin sections (8 μm) were obtained on a rotary microtome (Leica RM2245) with glass knives. The sections were always transferred alternately to three silanized microscopic slides, resulting in three almost identical series of sections. The first series was stained with hematoxylin and eosin for morphological evaluation, the second series was used for FISH with the probes for *Sodalis* and *Arsenophonus*, and the third was used for FISH with the probes for *Wolbachia* and *Rickettsia*. Samples were hybridized for 90 min at 50°C in hybridization buffer (0.9 M NaCl, 0.02 M Tris-HCl, pH 8.0, and 0.01% SDS) containing 25 nM each of the symbiont-specific probes as well as 5 μg/mL DAPI (4′,6-diamidino-2-phenylindole) for counterstaining of host cell nuclei. Residual probes were removed by a 20-min wash step at 50°C with prewarmed wash buffer (0.1 M NaCl, 0.02 M Tris-HCl, pH 8.0, 0.01% SDS, and 5 mM EDTA), followed by a 2-min washing step in distilled water (dH_2_O). After short rinsing in dH_2_O and shaking off the excess liquid, slides were covered with VectaShield H-1400 (Vector, Burlingame, USA) and inspected on an AxioImager Z2 fluorescence microscope with Apotome (Zeiss, Jena, Germany). The probes’ sequences were 5′-Cy5-CCGGCATTACCCGCTGGCAA-3′ for *Rickettsia* (59), 5′-Cy3-CTTCTGTGAGTACCGTCATTATC-3′ for *Wolbachia*: (59), 5′-Cy3-TCCGCTGACTCTCGCGAGAT-3′ for *Sodalis* (this study, modified from reference 60), and 5′-Cy5-CCTTAACACCTTCCTCACGAC-3′ for *Arsenophonus* (61). All probes targeted the 16S rRNA. Aposymbiotic S. cameroni (generated by antibiotic treatment) was used as negative control.

### Data availability.

The data that support the findings of this study are available in the supplemental material of this article.
