# Supplementary material for: Abundance and Localization of Symbiotic Bacterial Communities in the Fly Parasitoid Spalangia cameroni
Source: Appl Environ Microbiol. 2022 Apr 14;88(9):e02549-21. doi: 10.1128/aem.02549-21 (PMC9088259; doi:10.1128/aem.02549-21)
Supplement: Supplemental file 1 — Table S1. Download aem.02549-21-s0001.pdf, PDF file, 0.09 MB [file aem.02549-21-s0001.pdf]

<u>Supplementary table 1</u>. Standard curves' parameters.

Target organism	Gene	Insert size	Plate no.	Serial dilution	Samples' concentration range Pg/µl	Approx. Copy number range	Efficiency (%)	R^2
Spalangia cameroni	*28S*	185	1	1:10	0.01-100	10^3-10^7	77	0.99
			2				77	0.99
			3				79	0.99
			4				92	0.99
Wolbachia	*wsp*	218	1	1:5	0.064-40	10^4-10^7	78	0.99
			2				92	0.99
			3				90	0.99
			4				90	0.99
Rickettsia	*gltA*	438	1	1:5	0.3-188	10^4- 10^7	68	0.99
			2				59	0.99
Sodalis	*ompA*	223	1	1:10	0.001-100	10^2-10^7	90	0.98
			2				92	0.98
Arsenophonus	*InfB*	442	1	1:10	0.001-10	10^2-10^6	97	0.98
